# Diagnosis and Management of Cognitive Concerns in the Oldest-Old

**DOI:** 10.1007/s11940-021-00665-5

**Published:** 2021-03-26

**Authors:** Candace Borders, Seyed Ahmad Sajjadi

**Affiliations:** 1 Department of Neurology, University of California, Irvine, CA USA; 2 Neurology and Pathology, University of California, Irvine, CA USA

**Keywords:** Oldest-old, Dementia, Cognition, Sensory deficit

## Abstract

**Purpose of review:**

The fastest-growing group of elderly individuals is the “oldest-old,” usually defined as those age 85 years and above. These individuals account for much of the rapid increase in cases of dementing illness throughout the world but remain underrepresented in the body of literature on this topic. The aim of this review is first to outline the unique contributing factors and complications that must be considered by clinicians in evaluating an oldest-old individual with cognitive complaints. Secondly, the evidence for management of these cognitive concerns is reviewed.

**Recent findings:**

In addition to well-established associations between impaired cognition and physical disability, falls, and frailty, there is now evidence that exercise performed decades earlier confers a cognitive benefit in the oldest-old. Moreover, though aggressive blood pressure control is critical earlier in life for prevention of strokes, renal disease, and other comorbidities, hypertension started after age 80 is in fact associated with a decreased risk of clinical dementia, carrying significant implications for the medical management of oldest-old individuals. The oldest-old are more likely to reside in care facilities, where social isolation might be exacerbated by a consistently lower rate of internet-connected device use. The COVID-19 pandemic has not only highlighted the increased mortality rate among the oldest-old but has also brought the increased social isolation in this group to the forte.

**Summary:**

Differing from the “younger-old” in a number of respects, the oldest-old is a unique population not just in their vulnerability to cognitive disorders but also in the diagnostic challenges they can pose. The oldest-old are more likely to be afflicted by sensory deficits, physical disability, poor nutrition, frailty, and depression, which must be accounted for in the assessment of cognitive complaints as they may confound or complicate the presentation. Social isolation and institutionalization are also associated with impaired cognition, perhaps as sequelae, precipitants, or both. Ante-mortem diagnostic tools remain particularly limited among the oldest-old, especially given the likelihood of these individuals to have multiple co-occurring types of neuropathology, and the presence of neuropathology in those who remain cognitively intact. In addition to the symptomatic treatments indicated for patients of all ages with dementia, management of cognitive impairment in the oldest-old may be further optimized by use of assistive devices, augmentation of dietary protein, and liberalization of medication regimens for risk factors such as hypertension.

## Introduction

Dementia continues to pose a daunting challenge, not just to the patients who suffer from the condition and their families but also to the health care system and global economy. In the USA in 2020 alone, dementia is estimated to account for more than $300 billion in insurance payments, as well as 18.6 billion hours of unpaid caregiving [1]. The advancements in medicine that we have enjoyed over the past century have resulted in a relatively rapid increase in life expectancy. These major successes have had the unfortunate consequence of a steady increase in the global prevalence of cognitive impairment—as age is the risk factor most closely associated with the development of dementia.

Among older individuals (age 65 and above), the fastest-growing group is the “oldest-old,” variably defined in the literature as those age 85 or 90 and above [2]. The number of Americans in this group is expected to triple by 2050; meanwhile, dementia incidence continues to increase exponentially even beyond age 90 [1, 2]. The oldest-old are more likely to sustain multiple types of neuropathology and this increased burden is associated with decreased cognitive function [3]. The oldest-old are also far more likely to develop non-Alzheimer’s pathologies [4].

Factors like higher prevalence of sensory deficits, frequent comorbidities leading to polypharmacy, and higher prevalence of social isolation, make the oldest-old a unique population, differing from the elderly individuals less than 85 years of age (the “younger-old”). These factors have broad implications for the natural history and management of dementia in this population, and can make the process of diagnosis and management challenging. Clinicians and family members alike must be attuned to these key differences in order to provide holistic and effective care. In this review, we aim to describe the special considerations required in the assessment of cognitive concerns in the oldest-old, as well as the principles of management thereof.

## Assessment

### Assessment: medical and social history

**Fig. 1:**
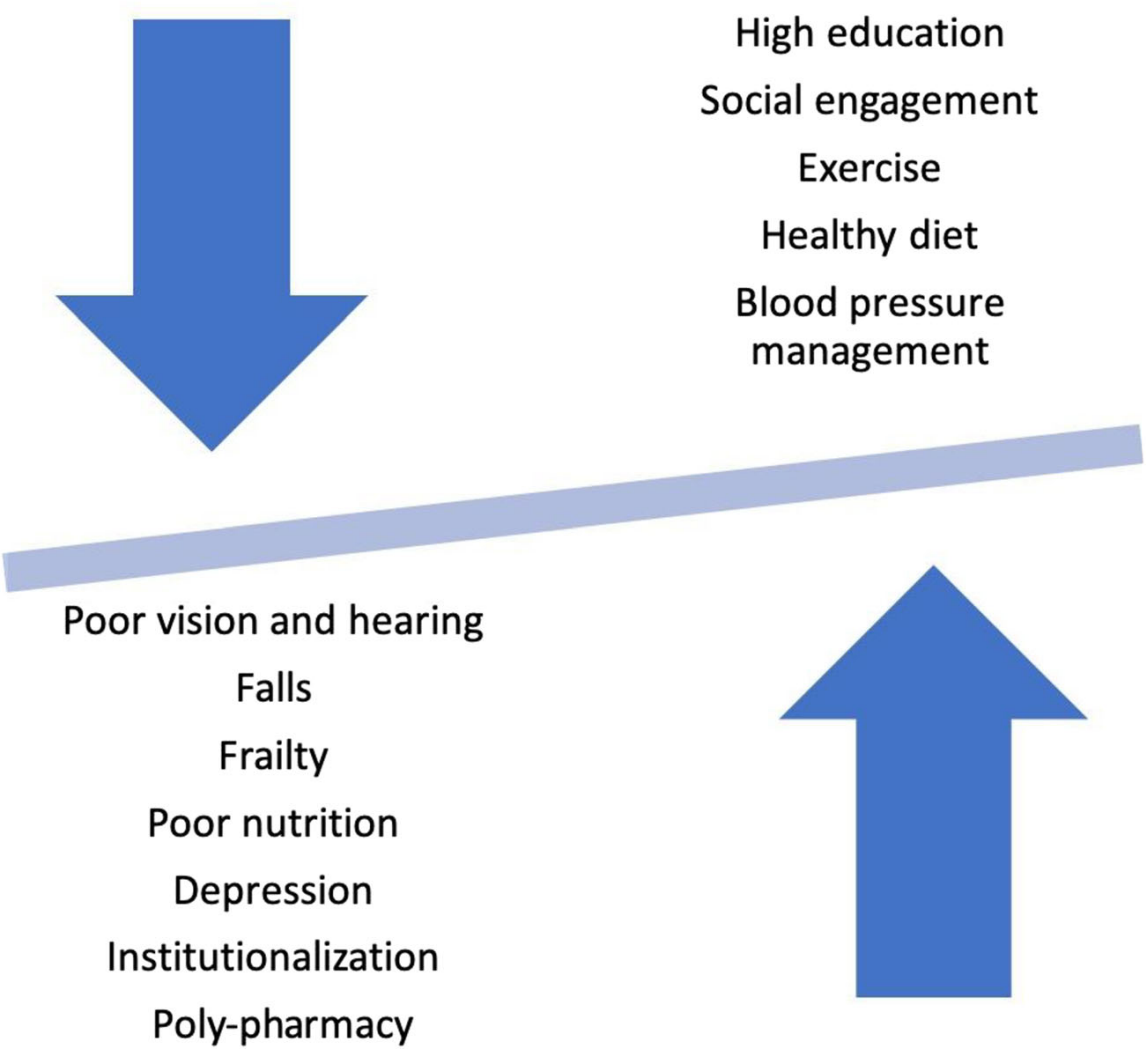
Schematic representation of factors helping and hindering cognition in the oldest-old.

As with patients of any age, clinical assessment of oldest-old individuals begins with discussion of the medical and social history. Making a diagnosis of cognitive impairment or dementia in the oldest-old is frequently challenging, as the functional deficits required for such diagnoses can also be caused or exacerbated by a number of comorbid medical and social circumstances (Fig. 1). Adequate evaluation of the oldest-old, therefore, requires heightened awareness of these differences.

#### Sensory deficits

One of the greatest challenges in assessing and caring for the oldest-old is also one of the most prevalent: the impaired transmission of sensory information from the outside world. Specifically, the oldest-old demonstrate pervasive deficits in hearing, vision, peripheral touch sensation, and vestibular function.

Age-related hearing loss, or presbycusis, is nearly universal among the oldest-old and in itself is an independent risk factor for incident dementia [5–8]. This may represent either a sequela of dementing illness or perhaps a contributing factor, due to the resulting difficulty in communicating and engaging with others. For instance, in a theoretical situation in which an individual only hears 50% of the spoken words, their “recall” of those spoken words will be 50% at best. Any memory difficulty will add to this apparent forgetting which is in fact due to impaired hearing function. Similarly, though some degree of age-related loss of visual acuity (presbyopia) is nearly universal in the elderly, there is evidence that the oldest-old suffer from a greater severity of vision loss [9, 10]. The association of vision loss with cognitive decline in the literature is murkier than that of hearing loss, with several cross-sectional studies showing a significant interaction [9, 11, 12] and others showing no relationship, especially when studied longitudinally [6, 12]. However, it is not difficult to imagine the correspondingly greater deficits in function and safety that this causes, even if cognition is intact. Visually impaired individuals are more likely to require assistance with activities of daily living, such as bathing and preparing meals—not to mention more complex tasks like driving and reading instructions on bottles of medication to administer [13]. Both hearing and vision impairment are also associated with depression and loneliness in the oldest-old [14, 15].

Sensory polyneuropathy of the distal extremities, affecting tactile pressure sensation as well as proprioception, increases with age and is present in approximately 30% of individuals over 80 [16, 17]. The vestibular system suffers age-related degeneration as well, both peripherally (due to the loss of otoconia, vestibular hair cells, and vestibular neurons) as well as centrally (due to reduced vertebrobasilar perfusion) [18]. Approximately half of individuals over 80 report daily balance problems, and in one small study of 38 individuals at least 85 years old, this proportion was nearly 70% [19]. These sensory impairments, combined with the aforementioned reduced visual acuity, can be thought of collectively as a syndrome of “presbyequilibrium,” the end result of which is postural instability, gait slowing, and an increased risk of falls [18–20].

#### Falls

Falls are associated with increased morbidity and mortality in the oldest-old, and the prevalence of falling increases with age [21–23]. In addition to the sequelae of falls directly related to impact, such as fractures and intracerebral hemorrhage, there are a number of other negative outcomes associated with falls in the oldest-old. Among these are the psychological effects of falling. In one study in the oldest-old with a mean participant age of 90, those who had fallen in the past year reported significantly lower self-confidence in balance, despite the absence of any difference in functional mobility between the two groups [24]. Similarly, in one longitudinal prospective study among a German oldest-old cohort, falls themselves (but not lower functional status) were associated with increased depressive symptoms [25].

#### Frailty

Frailty refers to the state of increased vulnerability to death and disability with even mild physiologic stressors [26]. It is therefore a multi-system concept involving the loss of muscle mass and strength (sarcopenia), immunodeficiency, and endocrine dysfunction [22, 27, 28]. The prevalence of frailty is 7% in the general elderly population but rises to at least 25% of individuals over 85 and 65% of those over 90 [26, 27, 29]. Frailty has been associated with cognitive impairment in the elderly and in the oldest-old specifically [30, 31]. Furthermore, it has been shown to be predictive of all-cause mortality in the oldest-old, independently or when co-occurring with cognitive impairment [27, 31].

#### Physical disability

Disability has been defined in the literature as having difficulty with and/or needing the help of another person to perform one or more of the following activities of daily living: bathing, toileting, dressing, transferring, walking indoors, and feeding. The prevalence of disability among individuals age 85 and older exceeds 50%, and the annual incidence after age 90 may triple in just 5 years, resulting in nearly universal disability among centenarians [32–35]. One longitudinal study in the oldest-old in China demonstrated a precipitous increase in disability prevalence in the 36 months prior to death [36]. Increased risk of disability in the oldest-old is associated with cognitive impairment, poor self-rated quality of life, and depression [32].

#### Social isolation

Across age groups, social engagement has been positively associated with general well-being, and in some studies with better physical health and reduced mortality [37–39]. Similarly, living alone and subjective loneliness are associated with depression in the elderly in general [15]. The oldest-old are uniquely vulnerable to lower levels of social engagement for a number of reasons. The majority of these individuals are widowed; they spend significantly more time alone; and are significantly less likely than other elderly adults to report having a confidant [32, 37, 40]. Medical illness and physical disability likely limit the degree of social engagement of which an individual is capable, and indeed the oldest-old with lower self-rated physical health report more social isolation [37]. The aforementioned cognitive and sensory deficits common among the oldest-old further limit their ability to leave their homes and to drive. Furthermore, as of 2018, less than 10% of individuals 80 years of age and older were estimated to use the internet at all, and a subsequent study in 2020 found that only 12% used a web-connected smartphone [41•]. Nearly all of the individuals accounting for this 12% were still residing at home. Web-connected device usage was almost nonexistent among those residing in facilities.

#### Physical environment

Although most oldest-old individuals reside in the community, it is perhaps unsurprising that the oldest-old comprise at least half of assisted living and nursing home residents [33, 42]. Relocation out of the home and into a facility ideally allows for greater supervision of those individuals who require it for safety but can also convey a host of detrimental effects. These include depressed mood, financial stress, loss of privacy, and loss of personhood [33, 43–45]. Older adults living in facilities where they are grouped by the level of care required, experience stigmatization and significant distress and may conceal health concerns due to a fear of involuntary relocation [43].

#### Nutrition

Malnutrition and unintentional weight loss in the elderly are multifactorial issues that worsen with age [46, 47]. The oldest-old are known to experience less anticipatory pleasure of meals, a necessary response to maintain appetite. [46] This may be a manifestation of well-established physiologic changes in the elderly including poor dentition, dry mouth, loss of olfactory and gustatory sensation, and slower gastric emptying [46, 47]. Other presumptive causes include a loss of involvement in meal preparation and feeding, as the oldest-old are more likely physically disabled and therefore reliant on a caregiver or meals-on-wheels service.

Skipping meals is more common in oldest-old individuals with depression, which is prevalent in this population (see below) [48]. The literature also suggests an association between poor nutritional status and dementia in the oldest-old that does not exist in cognitively normal or mildly cognitively impaired oldest-old individuals, even though there was no significant difference in the degree of medical comorbidity between these groups [49]. Lastly, one longitudinal prospective study demonstrated that malnutrition and underweight status were predictors of mortality among oldest-old Taiwanese individuals with recently diagnosed Alzheimer’s disease [50].

#### Depression

Most studies have found that the oldest-old are at increased risk of depression compared to other elderly individuals [15, 45, 51]. A notable exception to this found that the rates of mood disorders were level between ages 75 and 84 and age 85+ and that both groups were less likely to suffer from these conditions than individuals age 65–74 [52]. However, this study was limited by the exclusion of individuals residing in facilities, and institutionalization has a known association with depression in the elderly. Other pertinent risk factors for depression in the oldest-old previously discussed here include sensory impairment, falls, physical disability, and social isolation [14, 15, 25, 32].

Cross-sectional studies demonstrate an association between depression and cognitive impairment in the oldest-old, but too few longitudinal studies have been performed in this population to adequately address whether depression represents a cause, prodrome, or sequela of cognitive impairment (or some combination of these) [13, 43]. Regardless, identifying depression in the oldest-old is of the utmost importance because it may manifest with decreased cognitive and functional ability in this population, thereby complicating the clinical evaluation of cognition [53, 54].

#### Informants 2

In the setting of cognitive impairment, it is common to obtain a medical and social history primarily from an informant rather than from the patient himself or herself. This introduces the potential for bias based on the informant’s cultural expectations regarding aging and ability. For instance, they may consider a certain degree of forgetfulness or physical disability normal in the elderly, or fail to recognize loss of recent memories as long as long-term memory is intact [34].

## Assessment: diagnostics

Making a clinical diagnosis of cognitive impairment in the oldest-old can be challenging, for the reasons described in the prior section. The diagnostic criteria themselves, however, are the same for all age groups. Clinical dementia is diagnosed when an individual demonstrates a degree of cognitive impairment not due to another medical condition or toxic/metabolic derangement that impairs their ability to perform their usual activities independently. Mild cognitive impairment and “cognitive impairment no dementia” (CIND) are two useful ways of describing impairment that does not limit independence. Neuropsychological evaluation, biomarker testing, and neuroimaging can be useful tools for identifying the etiology of dementing illness (or illnesses) underlying a clinical diagnosis of cognitive impairment. However, the presence of multiple distinct neuropathologies in this population, among other things, affects the utility of such testing.

### Neuropsychological assessment

Historically, the administration of lengthy neuropsychological batteries to oldest-old individuals has been challenging for patients and clinicians alike, as these individuals are more susceptible to testing fatigue and frustration [55]. The results of such batteries are also more difficult to interpret in this population, as the oldest-old are either grouped with younger-old individuals in testing norms, or given separate norms with prohibitively small sample sizes [52]. Two reports based on The 90+ Study, a longitudinal study of dementia and its risk factors in the oldest-old in Laguna Woods, CA, provide the largest cohorts from which oldest-old normative data have been calculated [54, 55]. These studies propose a shorter neuropsychological testing battery comprised the following:

- Modified Mini-Mental State Examination
- Boston Naming Test, Animal Fluency Test, and Letter Fluency Test
- California Verbal Learning Test II
- Trail making test A, B, and C
- Clock Drawing Test
- CERAD Construction Test
- Wechsler Adult Intelligence Scale (Third Edition) Digit Span Test

In addition to those with cognitive impairment, cognitively intact oldest-old individuals in these two studies demonstrated a decline in performance on this battery with age [54, 55]. This decline may be attributable to increased sensory deficits, depression, or subclinical neurodegenerative pathology, but it was not associated with increased medical comorbidity.

### Serological markers

Commonly used serological biomarkers for AD include decreased levels of amyloid β_1–42_ and increased levels of total and phosphorylated tau in the cerebrospinal fluid (CSF). A CSF profile consistent with AD is detected in up to 36% of cognitively intact individuals by the age of 85, potentially limiting the utility of these studies [56]. This is in tandem with autopsy studies that have shown that over 40% of those who die without dementia in this age group had enough AD pathology to meet the neuropathological criteria for AD [57]. Additionally, since the oldest-old are more likely to have multiple neuropathologies producing the phenotype of dementia, the presence of these biomarkers does not necessarily exclude non-AD contributions [2].

### Positron emission tomography

As with serological markers, the value of positron emission tomography (PET) imaging is likely to be limited in the oldest-old across a number of targets. Consensus recommendations support the use of fluorodeoxyglucose (FDG) PET as a diagnostic tool in differentiating dementing illnesses in cases of atypical presentations, given its high negative predictive value for nonspecific neurodegeneration. However, the data comprising the basis for this recommendation involves a wide range of age groups and there is a significant paucity of data specific to the oldest-old on this topic [58]. Tau-PET imaging is similarly lacking data in oldest-old cohorts, and across age groups, its utility is limited by high cost without demonstration of consistently high sensitivity or specificity for dementia [59, 60]. Positive amyloid PET burden in non-demented oldest-old individuals has been associated with a lower cognitive baseline and steeper decline in cognitive functioning at 18-month, 24-month, and 12-year follow-up periods, although the clinical implications of this relationship for diagnosis and management of dementia are yet to be established [61–63].

### Magnetic resonance imaging

Brain magnetic resonance imaging (MRI) allows for visualization of hippocampal volume loss and subcortical white matter lesions (WMLs) due to microvascular ischemic change, as part of a standard work-up for cognitive concerns. These two findings are present in 32% and 26% of cognitively normal oldest-old individuals, respectively [61]. However, when co-occurring with +amyloid PET burden or with one another, they provide a significant predictor of cognitive impairment at 2-year follow-up [61]. Hippocampal atrophy alone was a significant predictor of cognitive impairment at 12-year follow-up [62]. In a corresponding study performed in an older sample (mean age 94) comprised both cognitively normal and impaired individuals, hippocampal atrophy and WMLs were independent of one another and associated with lower cognitive function at baseline and both were independent risk factors for greater cognitive decline at 2-year follow-up [64].

### Apolipoprotein E genetic status

Apolipoprotein E (*APOE*) status is considered one of the most important risk factors for the development of amyloid pathology. Across age groups, *APOE* ε4 carriers are considered to be at greater risk of developing β-amyloid neuropathology as well as clinical dementia, while *APOE* ε2 carriers are protected against dementia by mechanisms that are generally unknown [65]. In the oldest-old, *APOE* ε2 has been associated with decreased risk of clinical dementia and absence of β-amyloid on PET imaging, but not a significant decrease in the neuropathologic burden [66, 67]. *APOE* ε4, meanwhile, was associated with increased pathologic burden of amyloid but not with amyloid PET positivity [66, 67]. Additionally, a small longitudinal study of healthy individuals over 85 found high *APOE* ε2 allele frequency at baseline which further increased among survivors at 10-year follow-up [68].

Among female oldest-old individuals who carry the *APOE* ε4 allele, those who reached age 80 without developing cognitive impairment were found to have higher cognitive baselines and better self-rated overall health compared to their cognitively impaired counterparts, suggesting that genetic status likely interacts with other predisposing factors in the pathogenesis of dementia [69].

## Management

**Fig. 2:**
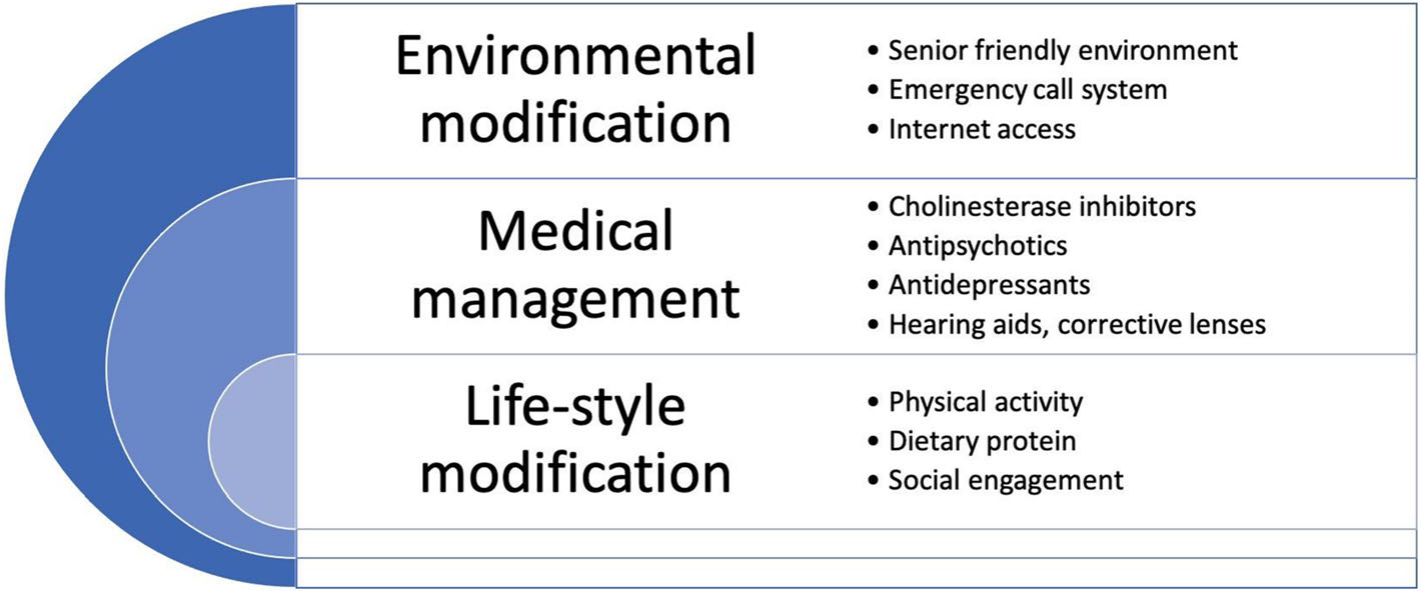
Multi-faceted management of problems related to cognition in the oldest-old.

Although treatment options for dementia and its complications remain very limited for all age groups, clinicians must be aware of pharmacologic and non-pharmacologic treatment recommendations, as well as the management of risk factors (Fig. 2). This is especially true for the oldest-old, who have the highest rates of these potentially modifiable risks.

### Symptomatic treatments

#### Cholinesterase inhibitors and NMDA antagonists

Indications for the use of symptomatic treatments in the oldest-old are the same as those for younger-old patients. However, it is worth noting that randomized controlled trials of cholinesterase inhibitors for the treatment of dementia due to Alzheimer’s disease (AD) have enrolled participants significantly younger than the mean age of actual patients with AD [70]. One study of 155 participants has suggested that cholinesterase inhibitors are no less safe or effective in the oldest-old with dementia compared to the younger-old with dementia [71]. The same study also included a single participant being treated with memantine, but further data on NMDA antagonists are lacking. Interestingly, one study found that donepezil is no more effective than *Ginkgo biloba* extract for the prevention of cognitive decline in the oldest-old [72].

#### Antipsychotics

Available studies regarding the use of antipsychotics in the oldest-old were conducted almost exclusively in institutionalized participants with high rates of dementia. Rates of antipsychotic use were high (16–45%) but lower than those of younger-old individuals in one study comparing the two groups [73–75]. Efficacy of antipsychotic treatment was not described in these studies.

#### Antidepressants

Rates of antidepressant use among the oldest-old with depression vary significantly across studies (0 to 60%), possibly correlating with the proportion of participants who are institutionalized [74–77]. In nursing homes, the oldest-old may be less likely than the younger-old to be treated with antidepressants [74, 77]. Data regarding the efficacy of antidepressants in the oldest-old is limited. One investigation of antidepressant use and completed suicide found that depressed individuals over 80 are significantly less likely than their counterparts age 50–59 to die by suicide when receiving antidepressant therapy [78]. Conversely, the individuals aged 80+ who did die by suicide were significantly less likely than the younger cohort to have been treated with antidepressants.

Lastly, there is evidence that light physical activity is inversely associated with depression in the oldest-old and that this relationship persists when controlling for medical comorbidities and level of cognitive functionality [79].

### Medical comorbidities

#### Cardiovascular risk factors

Oldest-old individuals are commonly treated with antihypertensive medications to prevent adverse sequelae of uncontrolled blood pressure, including hypertensive nephropathy, coronary artery disease, and ischemic and hemorrhagic stroke. There is, however, emerging evidence that cognition in the oldest-old may actually be harmed by aggressive lowering of blood pressure: individuals who were diagnosed with hypertension after age 90 were less likely than those age 80–89 to have clinical dementia at 3-year follow-up in one such study [80••]. In neuropathological assessments, hypertension was associated with larger hippocampal volume in one cohort of oldest-old individuals [63].

Congestive heart failure (CHF), on the other hand, is an established risk factor for cognitive impairment. The mechanism by which this occurs is postulated to be reduced brain perfusion as a result of declining cardiac pump function [81]. One study found that elevated brain natriuretic peptide (BNP) levels, a biomarker of CHF severity, was significantly associated with lower MMSE scores across 5-year follow-up visits [81]. The same study showed that concomitant elevated BNP with decreased systolic blood pressure was particularly disadvantageous, as these participants had significantly worse cognitive baselines and steeper decline. Though lower systolic BP may simply be due to worse cardiac pump function itself, this association may lend further support to the approach of treating hypertension less aggressively in the oldest-old. Moreover, a high degree of variability in systolic blood pressure was found to be associated with lower MMSE scores and increased WMLs in individuals 80 and older [82].

Dyslipidemia, including hypertriglyceridemia and hypercholesterolemia, is a well-established target for primary and secondary prevention of cardiovascular and cerebrovascular disease in middle-aged and younger-old adults [83, 84]. Statin prescription rates range from 12 to 59% in community-dwelling individuals at least 80 years of age, despite a relative lack of evidence for efficacy in this population [84]. In fact, low cholesterol levels in the oldest-old have been linked to increased mortality risk from cancer, trauma, and lung disease [83]. In one longitudinal study of individuals from age 70 to 90, the prevalence of hypercholesterolemia decreased with age, and statin use was associated with a survival benefit between ages 78 to 85 and 85 to 90 [85]. However, this reduction in mortality was found to be independent of serum cholesterol levels [85]. Moreover, among Chinese oldest-old individuals, higher serum triglyceride concentration has been associated with significantly lower risk of cognitive decline during a 5-year follow-up period. Together, these studies illustrate the need for further large-scale studies to define appropriate dyslipidemia treatment goals in the oldest-old.

#### Polypharmacy

The proportion of oldest-old individuals taking six or more medications varies in the literature from 30 to 70% [86–88]. High rates of polypharmacy are associated with non-adherence and adverse drug reactions across age groups, and with falls and cognitive impairment specifically in the elderly. De-prescribing of benzodiazepines in particular may offer one way for clinicians to better assess and treat cognitive concerns in the oldest-old [86, 87]. Meanwhile, there is some evidence that antithrombotic agents for secondary stroke prevention are actually under-prescribed in the oldest-old. This might be explained by clinicians’ concerns of potentially life-threatening bleeding after trauma due to falls [88]. However, this consideration must be balanced against the risk of allowing ischemic lesions to continue to accumulate, thereby worsening cognitive function.

### Lifestyle modification

#### Exercise

There is insufficient evidence for the effect of exercise in relation to dementia in the oldest-old, but there is evidence for this relationship in broader studies of adult or younger-old cohorts [27, 89]. However, exercise performed earlier in life does appear to confer increased protection against falls during the oldest-old time period [90••]. Moreover, falls risk-reduction programs administered to participants older than 85 have been shown to increase confidence and decrease the fear of falling, thereby increasing the amount of physical activity these individuals perform [91].

#### Nutrition

Among high-socioeconomic-status oldest-old men in Finland, healthy diet choices and vitamin D supplementation appeared protective against falls. [19] However, in the elderly as a whole, calcium and vitamin D intake have not consistently been associated with decreased incidence of fractures in the literature [92].

Dietary protein has been widely studied among the oldest-old as a potential target for mitigating sarcopenia. Increased protein intake has been positively associated with walking speed, grip strength, and ability to live independently in the oldest-old [93]. These associations could in turn have a beneficial effect on cognition, perhaps by reducing physical disability and institutionalization.

#### Alcohol and smoking

In younger-old cohorts, alcohol consumption and smoking have been associated with a higher risk of dementia; however, this relationship is not borne out when examined in the oldest-old exclusively [89]. A potential explanation is low frequency and amount of consumption in this age group.

#### Social engagement

Engaging in social or mental activities show a negative association with dementia in the oldest-old and could therefore be protective [89].

### Assistive devices and environment modification

Hearing aid use in adult and younger-old cohorts is positively associated with cognitive function, and this persists when controlling for depression and social isolation [94]. Among the oldest-old, these data are limited. The prevalence of hearing aid use in the oldest-old is approximately 30% [39]. Since presbycusis affects more than 30% of the oldest-old, it stands to reason that greater adoption of this assistive device might have an effect on the functional ability of these individuals, and possibly cognition itself. Mirroring the data on hearing impairment, the adequate correction of refractive errors limiting visual acuity is associated with better cognitive function, but these data were obtained in a predominantly younger-old cohort [95]. It is commonly recommended that visually impaired and/or elderly individuals maintain adequate lighting in the home and remove objects such as rugs in order to prevent mechanical falls, but data regarding the efficacy of these modifications is minimal.

Use of assistive devices for ambulation is associated with falls in the oldest-old, presumably due to the fact that individuals who fall are likely to be provided with these implements for safety [90••]. Wheelchair use and the presence of an emergency call system are similarly more prevalent in the institutionalized oldest-old, likely related to the higher degree of physical disability that required institutionalization in the first place [39].

### COVID-19 considerations

The oldest-old are at particular risk with regard to COVID-19. In Lombardy, Italy, the percentage increase in mortality in March 2020 compared to 2019 was more noticeable with advancing age with a remarkable level of granularity—individuals were grouped in 5-year increments of age all the way up to and including centenarians [96]. In a subgroup analysis of 91 oldest-old individuals with COVID-19 in Wuhan, China, these individuals were found to have significantly greater risk of extrapulmonary sequelae of the disease and greater mortality compared to their younger-old counterparts [97].

A recent study in Maine-et-Loire, France, tested every resident of a single nursing home for individuals with major neurocognitive disorders (mean age 88) [98•]. Symptoms present in the 47% testing positive for infection were less likely to include fever and cough and more likely to include acute cardiac injury and nonspecific encephalopathy and anorexia compared to younger cohorts in other studies, possibly related to overall poorer baseline nutritional status. This may support the implementation of COVID-19 testing en masse in this age group.

Staying home is clearly of the utmost importance in protecting the oldest-old from COVID-19, but it is worth noting that this could have long-term consequences for cognition and mood by worsening isolation and eliminating opportunities for exercise. A recent article has emphasized the importance and efficacy of simple home exercises in maintaining functionality in the oldest-old, especially since these do not compromise social distancing efforts [99].

## Conclusion

The oldest-old is a unique, and uniquely vulnerable, population with regard to cognitive impairment and its risk factors. The relationship of medical comorbidities and social circumstances in the oldest-old to cognition cannot be overlooked by treating clinicians. Although ante-mortem biomarkers for dementing illnesses are wanting across all age groups, there is a growing body of research in this area among the oldest-old. Neuropathologic studies in this population have yielded a number of findings that will ultimately help define the pathogenesis of dementing illnesses, with the goal of identifying therapeutic targets in the future. Non-pharmacologic interventions and management of comorbidities allow further opportunities to alleviate the burden of cognitive impairment experienced by these individuals and felt by their families and caregivers. All of these measures together are necessary to maintain health, dignity, and safety in our rapidly aging population.
